# Back Numbers

**Published:** 1877-03

**Authors:** 


### Back Numbers.

December, January and February numbers of the JOURNAL have not been published and cannot now be published in time to be respectable. As we are wholly unwilling to publish a journal late, we shall omit them altogether and deduct their value from the price of Vol. XVI. Hereafter we shall issue our journal regularly and promptly. If it is not received, it may be assumed that the Post Office department of the United States is at fault, and we want notice of the failure.

